# Mineralizing Gelatin Microparticles as Cell Carrier and Drug Delivery System for siRNA for Bone Tissue Engineering

**DOI:** 10.3390/pharmaceutics14030548

**Published:** 2022-02-28

**Authors:** Sandra Hinkelmann, Alexandra H. Springwald, Sabine Schulze, Ute Hempel, Franziska Mitrach, Christian Wölk, Michael C. Hacker, Michaela Schulz-Siegmund

**Affiliations:** 1 Pharmaceutical Technology, Faculty of Medicine, Institute of Pharmacy, University of Leipzig, Eilenburger Straße 15A, 04317 Leipzig, Germany; sandra.hinkelmann@medizin.uni-leipzig.de (S.H.); alexandra.springwald@uni-leipzig.de (A.H.S.); franziska.mitrach@medizin.uni-leipzig.de (F.M.); christian.woelk@medizin.uni-leipzig.de (C.W.); michael.hacker@hhu.de (M.C.H.); 2 Joint and Soft Tissue Research, Centre for Translational Bone, Faculty of Medicine, TU Dresden, Fiedlerstraße 34, 01307 Dresden, Germany; sabine.schulze@tu-dresden.de; 3 Institute of Physiological Chemistry, TU Dresden, Fiedlerstraße 42, 01307 Dresden, Germany; ute.hempel@tu-dresden.de; 4 Institute of Pharmaceutics and Biopharmaceutics, Heinrich Heine University, Universitätsstr. 1, 40225 Düsseldorf, Germany

**Keywords:** chordin silencing, human mesenchymal stem cells (hMSC), human peripheral blood mononuclear cells (hPBMC), gelatin microparticles, osteogenic differentiation, co-culture

## Abstract

The local release of complexed siRNA from biomaterials opens precisely targeted therapeutic options. In this study, complexed siRNA was loaded to gelatin microparticles cross-linked (cGM) with an anhydride-containing oligomer (oPNMA). We aggregated these siRNA-loaded cGM with human mesenchymal stem cells (hMSC) to microtissues and stimulated them with osteogenic supplements. An efficient knockdown of chordin, a BMP-2 antagonist, caused a remarkably increased alkaline phosphatase (ALP) activity in the microtissues. cGM, as a component of microtissues, mineralized in a differentiation medium within 8–9 days, both in the presence and in the absence of cells. In order to investigate the effects of our pre-differentiated and chordin-silenced microtissues on bone homeostasis, we simulated in vivo conditions in an unstimulated co-culture system of hMSC and human peripheral blood mononuclear cells (hPBMC). We found enhanced ALP activity and osteoprotegerin (OPG) secretion in the model system compared to control microtissues. Our results suggest osteoanabolic effects of pre-differentiated and chordin-silenced microtissues.

## 1. Introduction

Since its discovery in 1998, the development of RNA interference (RNAi) strategies in regenerative medicine have progressed rapidly [1,2,3,4,5]. The RNAi mechanism enables a targeted and post-transcriptional silencing of gene expression by small interfering RNA (siRNA) and microRNA (miRNA) [6,7,8]. Both can bind and induce the degradation of complementary messenger RNA (mRNA) by activating the RNA-induced silencing complex (RISC) [9]. Previous studies have shown that RNAi can improve bone regeneration in vitro [10,11,12,13] and in vivo [14,15]. While the use of siRNA allows a very specific downregulation of proteins that control cell differentiation, miRNAs usually have several targets, with a multitude of consequences to cellular processes. Nonetheless, the application of siRNA is limited by natural barriers, such as nucleases present in the tissue able to quickly degrade naked siRNA and an inefficient cellular uptake due to the anionic character and large molecular weight of siRNA [16,17,18]. To overcome these limitations, a broad variety of transfection reagents (e.g., cationic polymers, cationic lipid composites, and peptides) have been developed that protect the siRNA by complex formation and enable cellular uptake as well as intracellular release [18,19]. Nevertheless, the type of application is still decisive for the biological outcome of a silencing strategy. While systemic application of siRNA complexes often leads to accumulation in the liver and provides immunostimulatory activity, the local application may increase the therapeutic effect [19,20]. Using siRNA-loaded hydrogels that mimic the extracellular matrix and present siRNA to co-encapsulated or attaching cells could be an interesting application strategy in regenerative medicine [21,22,23,24].

In this project, we focused on an innovative approach for the regeneration of bone tissue, to meet the clinical need for strategies to replace autologous bone grafts. Although the transplantation of autologous bone is still the gold standard in bone repair, the available amount is limited, depending on the existing bone structure and underlying diseases of the patient [25]. However, we think that the use of stem cells instead of whole tissue fragments may suffice to stimulate regeneration in defective bone tissue [26,27]. For this reason, we aggregated human mesenchymal stem cells (hMSC) with siRNA delivering cross-linked gelatin microparticles (cGM) to form microtissues for the treatment of bone defects. Microtissues were additionally stimulated with osteogenic supplements. These 3D constructs mimic a biosimilar microenvironment with cell–cell and cell–matrix contacts. The microparticles provide a substrate for cell adhesion and improve oxygen and nutrient supply in the microtissues [28,29]. In order to enhance bone regeneration by siRNA, we silenced chordin, an antagonist of the bone morphogenetic protein 2 (BMP-2) [30,31], and followed the hypothesis that the silencing of chordin in combination with BMP-2 supplementation may enhance osteogenic differentiation and accelerate mineralization in osteogenic cultures [10,13,32,33,34]. These microtissues may be applied similarly as autologous bone grafts.

In a previous study, we reported on non-mineralizing gelatin microparticles and showed improved mineralization of the chordin siRNA-releasing microparticles [34]. Here, we changed the protocol to greatly increase the cross-linking degree of cGMs from 37.2% to 50.7%. As a decisive consequence of the stronger cross-linking reaction between the anhydride-containing (oPNMA) and amino groups in gelatin, the number of carboxylate groups in the microparticles is concomitantly increased. The increased anionic charge density is expected to improve calcium affinity, which has been described as a nucleation center for mineral formation [35]. Via partial reaction of oPNMA with *N, N*-diethylethylenediamine (DEED), we derivatized the cross-linker with non-reactive tertiary amine functionalities. The changed electrostatic properties are designed to stabilize complexed siRNA and make it available for cellular uptake. This study aimed to investigate if the stronger cross-linking affects hydroxyapatite (HA) formation as the mineral phase of bone tissue in the presence and absence of cells. Further to this, we investigated if the higher cross-linking degree had an influence on the silencing efficiency of the chordin siRNA loaded to the cGM. Finally, we tested the effects of microtissues on bone homeostasis in a co-culture model of hMSC and human peripheral blood mononuclear cells (hPBMC). Here, we were able to show a high silencing effect due to the changed electrostatic properties of the particle surface in contrast to the previously published data [34].

## 2. Materials and Methods

### 2.1. Particle Fabrication

Gelatin microparticles (GM) cross-linked with the pre-derivatized oligomeric cross-linker oligo (pentaerythritol diacrylate monostearate (P)-*co*-*N*-isopropylacrylamide(N)-*co*-maleic anhydride (MA)) (oPNMA-7.5^+DEED^) were fabricated according to published protocols [34,36,37]. Compared to the protocol published before [34], cross-linking degree of the cGM was increased by addition of triethylamine (TEA). Briefly, 1 g of GM was dispersed in 2 mL of acetone before the pre-derivatized reaction solution containing oPNMA-7.5^+DEED^ as cross-linker, 2 mL of the base triethylamine (TEA), and 10 mL of water were added, stirred for 4 h, then washed and dried as described previously [34]. The particles were characterized as described in the prior publication [34].

### 2.2. Preparation of siRNA-Loaded Microparticles

For loading of the microparticles, 703.13 ng chordin siRNA (Dharmacon, Lafayette, CO, USA) and 15.78 µL Lipofectamine^®^ RNAiMAX (Thermo Fisher Scientific™, Schwerte, Germany) were complexed in 24 µL, added to 3 mg dried cGM, vortexed and incubated at 4 °C for 30 min [34]. Subsequently, loaded cGM were resuspended in Opti-MEM™ (Thermo Fisher Scientific™, Schwerte, Germany) supplemented with 10% FBS. The final suspension contained 0.064 mg cGM loaded with 15 ng siRNA/100 µL. As control, cGM were loaded with Opti-MEM™ only (ctr: non-treated control) or All Stars Negative Control (Qiagen, Hilden, Germany) siRNA (nc: non-coding control).

### 2.3. Microtissues

Assembling of cells and particles to microtissues followed a previously described protocol [34]. Briefly, hMSC (Lonza, Basel, Switzerland) were cultivated in DMEM low glucose (Sigma Aldrich, Seelze, Germany), 10% (*v/v*) fetal bovine serum albumin (FBS, Biochrome, Berlin, Germany), 1% amino acids and antibiotics and detached the cells with Trypsin/EDTA (Sigma Aldrich, Seelze, Germany). We added 100 µL of cell suspension (10^5^ cells ml^−1^) to 100 µL of cGM suspension (0.64 mg ml^−1^) in Opti-MEM™ with 10% FBS per well of a low adhesion 96-well plate (Spheroid microplate, Corning, NY, USA). After 24 h, the medium was changed to osteogenic medium (OM) based on DMEM, 10% (*v/v*) FBS, 100 ng/mL Dexamethasone (Sigma Aldrich, Seelze, Germany), 50 µg/mL ascorbic acid (Sigma Aldrich, Seelze, Germany), 10 mM β-Glycerophosphate disodium salt hydrate (Sigma Aldrich, Seelze, Germany) with (OM^+BMP-2^)/without (OM) 100 ng/mL bone morphogenetic protein (BMP-2, R&D Systems, Wiesbaden, Germany). Medium was changed partially by exchanging 200 µL of culture medium twice for three times a week in order to keep cGM/hMSC and microtissues in the well.

### 2.4. Gene Expression Analyses

RNA isolation, cDNA synthesis, and PCR were performed as previously described [34]. Used TaqMan™ gene expression assays are shown in Table 1. 60S acidic ribosomal protein P0 (RPLP0) served as a housekeeping gene [38]. 

### 2.5. Osteogenic Differentiation of hMSC in Microtissues

Alkaline phosphatase (ALP) activity and calcium content were determined and normalized to deoxyribonucleic acid (DNA) content in the microtissues as previously published [34]. For calculation of Ca/P-ratio, phosphate concentration was measured with a phosphorus quantification kit (Greiner Diagnostic Group, Bahlingen, Germany). DNA quantity was determined for normalization of ALP activity and calcium content. DNA quantity in microtissues was measured in a Plate Reader Synergy H1 (BioTek, Bad Friedrichshall, Germany) using Quant-iT™ PicoGreen™ dsDNA (Invitrogen™, Darmstadt, Germany) following a previously described protocol [10,11]. A standard curve of increasing DNA dilutions (Invitrogen™, Darmstadt, Germany) in a range of 0.001–10 µg/mL was used for the quantification of unknown samples.

### 2.6. Cryosectioning

Samples were washed with warm PBS (37 °C), fixed with 10% paraformaldehyde (PFA) for 1 h at RT. Dehydration of samples was conducted with 30% sucrose for 24 h (4 °C). Then, samples were embedded in Tissue Freezing Medium (Leica, Wetzlar, Germany) at −80 °C for 15 min. Frozen sections of 10–20 µm thickness were prepared by Cryostat (Leica, Wetzlar, Germany).

### 2.7. Microscopy

Washed and PFA fixed samples were stained with OsteoImage (Lonza, Basel, Switzerland) for visualization of hydroxyapatite (HA), as reported before [34], according to the manual. Staining with 1 µL 4′,6-diamidino-2-phenylindole (DAPI) and 25 µL Alexa Fluor™ 568 phalloidin (Thermo Fisher Scientific™, Schwerte, Germany) was performed to analyze cell morphology. Alizarin Red staining was performed to visualize matrix formation and mineralization in cryosections as reported before [34]. A confocal microscope Leica TCS SP8 and LAS X software (Leica, Wetzlar, Germany) were used for imaging.

### 2.8. Microtissues in a Supplement-Free Co-Culture System of hMSC and PBMC

Peripheral blood mononuclear cells (PBMC) and hMSC were directly co-cultured with microtissues on an osteoblast-derived and decellularized extracellular matrix generated by SaOS-2 cells. The experimental procedure followed previously described protocols [39,40]. In brief, microtissues were assembled with hMSC as described above and incubated for 7 days in OM or OM^+BMP-2^. PBMC were isolated from donated buffy coats by Ficoll gradient centrifugation and seeded (1.5 × 10^5^/cm^2^) on the matrix in α-MEM (Sigma Aldrich, Seelze, Germany) with 20% heat-inactivated FBS, 1% P/S, and 2 mM glutamine (Sigma Aldrich, Seelze, Germany). hMSC suspension (2500/cm^2^) was added after 2 h and pre-differentiated microtissues after another 30 min. Two microtissues were placed at the edges of one well. The supernatants were collected after 3, 6, 10, and 14 days and analyzed with Human Osteoprotegerin DuoSet Enzyme-linked immunosorbent assay (ELISA, R&D Systems, Wiesbaden, Germany). ALP and tartrate-resistant acid phosphatase (TRAP) were analyzed after 14 days as previously described [40]. Adsorption values were calculated concerning non-treated cells in OM and set to 1 as control.

### 2.9. Statistics

One-way ANOVA with Tukey’s post hoc test was performed using GraphPad Prism version 9.2.0 for Windows (GraphPad Software, La Jolla California, CA, USA). Statistically significant differences between groups (*p* < 0.05) are indicated by (*), (#), or (§).

## 3. Results

### 3.1. Characterization of Mineralizing oPNMA-7.5^+DEED^ Cross-Linked Gelatin Microparticles

oPNMA-7.5^+DEED^ cGM were obtained as a free-flowing, dry powder with a cross-linking degree of 50.7% and an average particle size of D [4,3] = 79.15 ± 10.68 µm in the dry state (Table 2). For PBS uptake, a factor of 10.8, representing the fold increase in wet weight over dry weight, was calculated. The average particle size of particles swollen in PBS overnight was D [4,3] = 137.67 ± 18.77 µm.

### 3.2. Silencing Efficiency of cGM-Released siRNA

**Figure 1:**
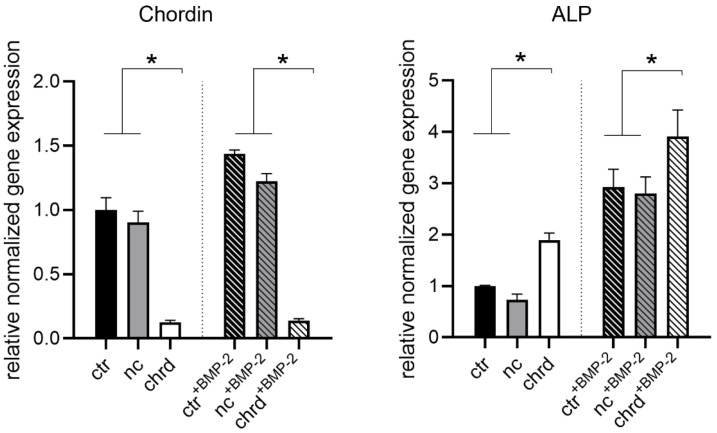
Chordin and alkaline phosphatase (ALP) expression in microtissues assembled from siRNA-loaded cGM and hMSC after 4 days in an osteogenic medium with or without BMP-2 (ctr: non-treated control, nc: non-coding siRNA, chrd: chordin siRNA). Statistically significant differences (*p* < 0.05) are indicated with (*).

The effect of siRNA released from cGM in microtissues was investigated in osteogenic media with or without BMP-2 by gene expression analysis after 4 days (Figure 1). We detected effective chordin silencing in both groups with statistically significant differences between chordin siRNA and non-treated or non-coding control. Additionally, a significant increase in ALP gene expression was measured already after 4 days when chordin siRNA was loaded to cGM in microtissues. This effect was enhanced when samples were stimulated with BMP-2.

**Figure 2:**
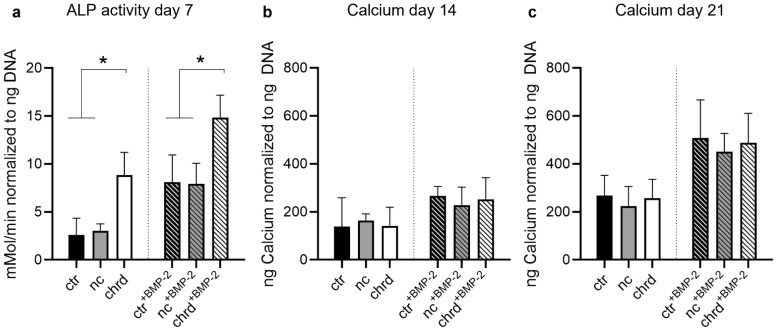
(**a**) ALP activity after 7 days, and mineralization after (**b**) 14 and (**c**) 21 in microtissues assembled from siRNA-loaded cGM and hMSC cultured in osteogenic medium with or without BMP-2 (ctr: non-treated control, nc: non-coding siRNA, chrd: chordin siRNA). Statistically significant differences (*p* < 0.05) are indicated with (*).

**Figure 3:**
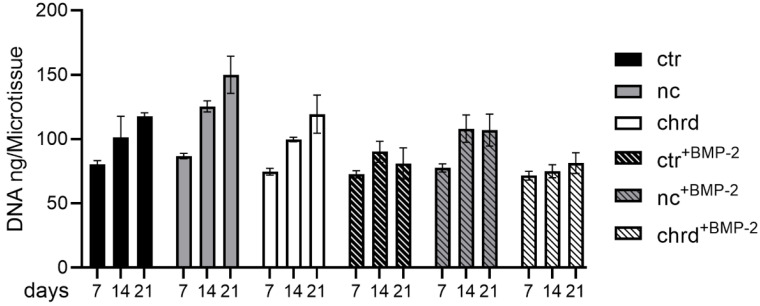
DNA content after 7, 14, and 21 days in microtissues assembled from siRNA-loaded cGM and hMSC cultured in osteogenic medium with or without BMP-2 (ctr: non-treated control, nc: non-coding siRNA, chrd: chordin siRNA).

In order to analyze downstream effects of chordin silencing in microtissues, ALP activity was measured after 7 days, and mineralization was quantified after 14 and 21 days. We detected a significantly increased ALP activity in microtissues when chordin siRNA was loaded to cGM (Figure 2a), which is in accordance with gene expression data. By BMP-2 stimulation, this effect was enhanced in comparison to non-stimulated or non-coding siRNA samples. However, we could not find significant differences in calcium quantities between chordin-silenced and non-silenced samples (Figure 2b and Figure S1). Nevertheless, a distinct increase in the amount of calcium was observed between 14 and 21 days in BMP-2-stimulated and non-stimulated samples. In order to analyze the proliferation in microtissues over 21 days, DNA content was determined on days 7, 14, and 21. The proliferation rate of hMSC in BMP-2-stimulated microtissues was strongly decreased, irrespective of chordin silencing (Figure 3). In the samples without BMP-2 stimulation, a linear increase in the DNA content could be measured in all groups (Figure 3 and Table 3).

### 3.3. Differences during Mineralization after Chordin Silencing

**Figure 4:**
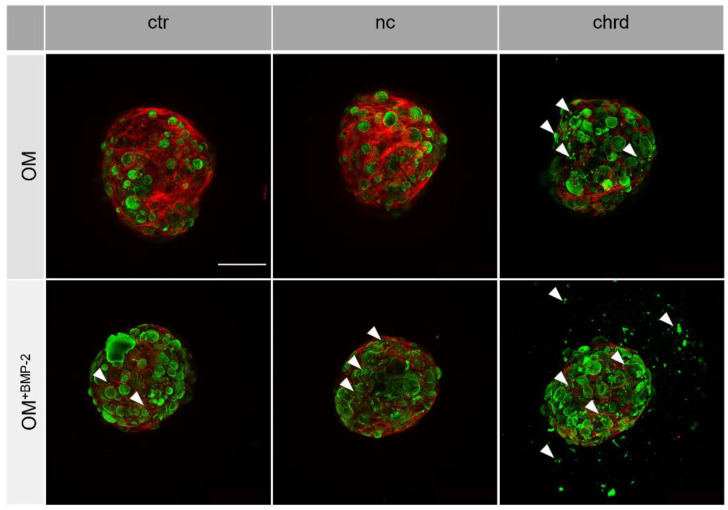
Representative images of hydroxyapatite (HA) formation (OsteoImage staining) in microtissues from hMSC (green: HA, red: actin cytoskeleton) with or without chordin siRNA by confocal laser scanning microscopy (HA formations in the interspace of agglomerated cGMs are highlighted by white arrows). Stem cells were cultivated in an osteogenic medium (OM) with or without BMP-2 for 21 days. Scale bar represents 500 µm.

We also analyzed the morphology of microtissues to obtain a better understanding of the mineral quality. OsteoImage staining (green) indicated the presence of hydroxyapatite (HA) in the mineralized cGM (Figure 4). The fluorescence signal in chordin-silenced microtissues was slightly increased in comparison to the non-coding or non-treated controls. BMP-2-stimulated microtissues generally showed a higher HA attributable fluorescence intensity than samples cultivated without BMP-2. In the chordin-silenced microtissues cultivated without BMP-2 and in all BMP-2-stimulated groups, we found extensive HA formation in the interspace of agglomerated cGMs (highlighted by white arrows). When chordin was silenced and microtissues were cultured with BMP-2, the HA-attributed fluorescence signal was even detectable at the surface of the surrounding well.

**Figure 5:**
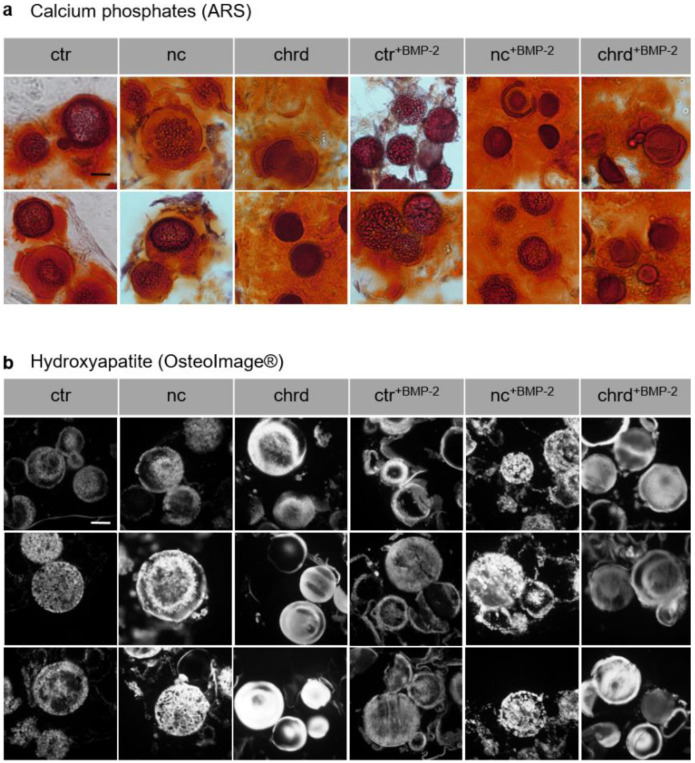
(**a**) Alizarin red staining (ARS) and (**b**) OsteoImage staining of cryosections (10–20 µm) of mineralized microtissues with and without siRNA or/and BMP-2 after 21 days in osteogenic medium (ctr: non-treated control, nc: non-coding siRNA, chrd: chordin siRNA). Scale bar represents 50 µm.

Alizarin red staining (ARS) of cryosections was carried out to visualize mineralized extracellular matrix components in general (Figure 5a) and OsteoImage staining was performed for the identification of HA structures (Figure 5b). Obvious differences in the surface structure of the cGM could be recognized in the cryosections. Microparticles in chordin-silenced microtissues showed a smooth structure at the cross-section area in the inner part, whereas rough, dotted HA structures were visible in the non-coding and to a lesser degree in the non-treated controls.

**Figure 6:**
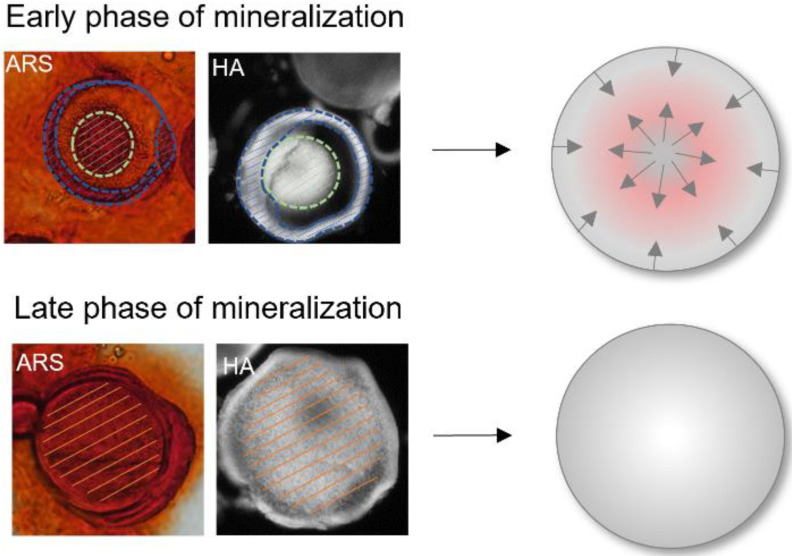
Close-up of cGM in the early and late mineralization phase stained with Alizarin Red staining (ARS) or OsteoImage (HA). The mineralized areas have been marked with dashed lines in green for the inner mineralization zone, in blue for the outer mineralization zone, and orange for completely mineralized particles.

**Figure 7:**
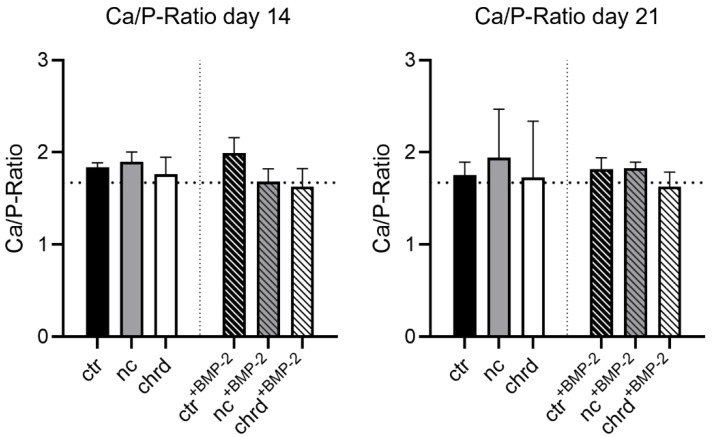
Ca/P ratio after 14 and 21 days (ctr: non-treated control, nc: non-coding siRNA, chrd: chordin siRNA) in microtissues from siRNA-loaded cross-linked gelatin microparticles (cGM) and hMSC in an osteogenic medium (OM) with or without BMP-2 (ctr: non-treated control, nc: non-coding siRNA, chrd: chordin siRNA). Dashed line: stoichiometric Ca/P ratio of pure hydroxyapatite (1.67).

Mineralization in the microparticles apparently started from two directions in all groups. In the early mineralization stages, ARS- and OsteoImage-positive structures were found in the inner core and the outer layer (Figure 6). In later stages of mineralization, minerals were detected throughout the entire cGM. In order to analyze mineralization quality, we determined the calcium–phosphate ratio in the mineralized samples (Figure 7). Chordin-silenced microtissues, which were stimulated with BMP-2 showed a close approximation to the stoichiometric value (1.67) of pure HA at both measuring points. The control groups showed values with a tendency to higher values than 1.67. However, the differences between the groups were not significant. For a better understanding of mineralization in cGM, a defined amount of cGM was placed in a spheroid microwell in the absence of cells and identically cultivated to the treatment of microtissues with cells. We observed mineralization of particles even in the absence of cells in the differentiation medium (Supplementary Materials Figure S2).

### 3.4. Pre-Differentiated Microtissues in a Supplement-Free Co-Culture of hPBMC and hMSC

**Figure 8:**
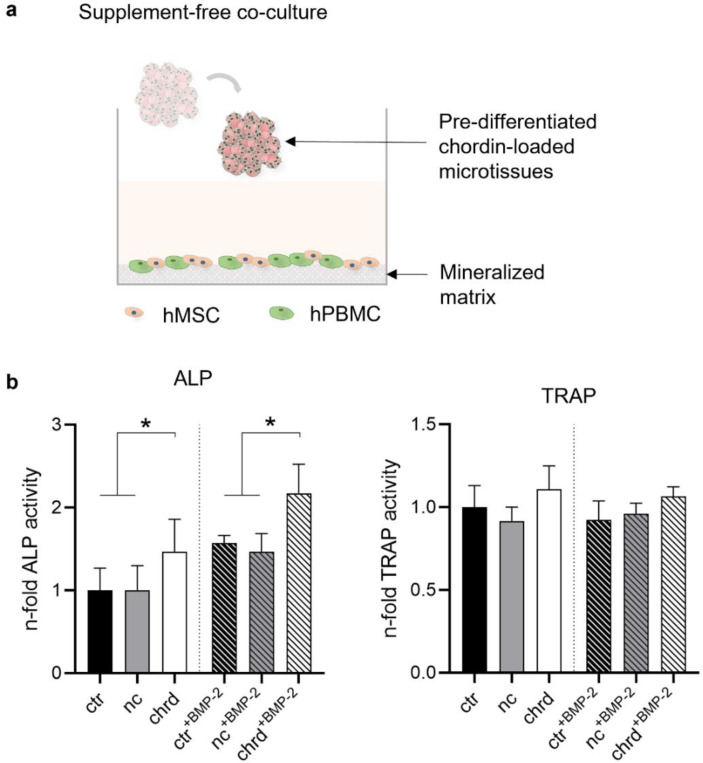
(**a**) Graphical outline of the experimental procedure: chordin siRNA-loaded microtissues were stimulated in OM or OM^+BMP-2^ for 7 days and transferred to a supplement-free co-culture system of hMSC (human mesenchymal stem cells) and hPBMC (human peripheral blood mononuclear cells) seeded on a decellularized mineralized matrix. hPBMC and hMSC were cultivated together with pre-differentiated microtissues in a supplement-free culture medium for 14 days. (**b**) ALP and TRAP activity were analyzed after 14 days of supplement-free co-culture (ctr: non-treated control, nc: non-coding siRNA, chrd: chordin siRNA). Statistically significant differences (*p* < 0.05) are indicated with (*).

For first investigations regarding the suitability of microtissues as a therapeutic approach for bone regeneration, we added pre-differentiated microtissues (7 days in OM or OM^+BMP-2^) to a co-culture system of undifferentiated hMSC and PBMC [39,40]. In preparation for the supplement-free co-culture of osteoblasts and osteoclasts, well plates were cultivated with SaOS-2 cells for ~25 days in an osteogenic medium to generate a homogeneous layer of the extracellular matrix with a reservoir of bone matrix mediators. That matrix was decellularized before hMSC and PBMC were seeded and cultured in basic culture medium without differentiating supplements. Two microtissues were placed at the edge of each well and co-cultured with hMSC and hPBMC for 14 days to determine the effects of microtissues on osteogenic and osteoclastic differentiation (Figure 8).

**Figure 9:**
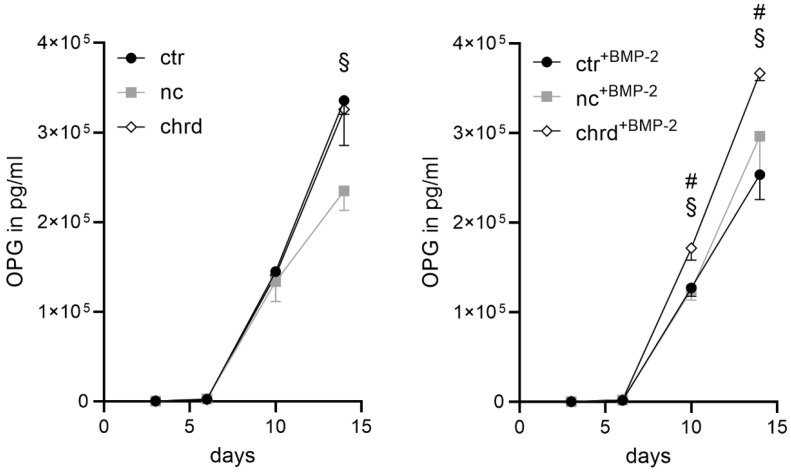
Osteoprotegerin (OPG) in the supernatant during supplement-free co-culture of hMSC (human mesenchymal stem cells) and hPBMC (human peripheral blood mononuclear cells) seeded on a decellularized mineralized matrix with pre-differentiated microtissues (ctr: non-treated control, nc: non-coding siRNA, chrd: chordin siRNA). Statistically significant differences (*p* < 0.05) between ctr and chrd are indicated with (#), between nc and chrd with (§).

Co-cultures with chordin-silenced microtissues pre-treated with and without BMP-2 showed significantly higher ALP activities compared to the non-treated and non-coding control. However, we could not find any differences in TRAP activity. In order to determine the interaction of osteoblasts and osteoclasts, osteoprotegerin (OPG) as a key player in the differentiation of osteoclasts was determined. OPG, secreted by osteoblasts, is a soluble receptor and an inhibitor of receptor activator of nuclear factor kappa B ligand (RANKL) [41,42]. Significantly increased OPG concentrations were found in the supernatant of the co-culture with silenced microtissues pre-treated with BMP-2 compared to all other groups on days 10 and 14 (Figure 9). Without BMP-2, the effect was only detectable between the non-coding control and the chordin-silenced group.

## 4. Discussion

This study continued a former study [34] where we established the microtissue formation and obtained the first hints that silencing via cGM is promising. The important difference to the previously published work is the clearly higher degree of cross-linking of the investigated cGMs. The degree of cross-linking was 50% compared to 37% in the former study as a result of the addition of TEA during the cross-linking reaction. That characteristic most probably increased the charge density at/in the GM, with negatively charged carboxy groups originating from the aminolyzed maleic anhydrides (MA) and positively charged amino groups from the partial DEED derivatization of the MA groups.

We developed stable microtissues from the combination of hMSC and siRNA-loaded microparticles, which in the future could be implanted directly into bone defects via minimally invasive surgery or embedded in a hydrogel or a fiber mesh. Our system offers the basis for a multi-unit system with options to modify the biophysical and biochemical properties [43] of cGM, the choice of siRNA target and complexation, and finally the cell type and cell pre-treatment. We could show in this study, that an apparently small difference as an increase in the cross-linking degree results in clearly improved silencing and mineral composition.

### 4.1. Effective Silencing by cGM-Released siRNA

It has already been described that microparticles incorporated in cell aggregates could improve the supply of nutrients and oxygen in the inner core and, at the same time, also release active ingredients in order to affect surrounding cells locally [21,28,29,43,44,45,46]. In our study, we could successfully demonstrate that the synthesized cGM serves as a cell carrier providing structural support and nutrient supply and as a drug delivery biomaterial for siRNA in microtissues. The local presentation of chordin siRNA to hMSC in the microtissues led to a chordin knockdown of approximately 85% compared to the non-treated and non-coding control. Non-complexed (naked) siRNA was not considered as a control as we did not find any silencing effects in former studies in accordance with the literature [17,18]. 

We believe that electrostatic interaction between the positively charged siRNA complexes and negatively charged carboxyl groups present in gelatin and introduced by the cross-linker are involved to effectively present and deliver the complexed siRNA directly to adhering cells [34]. Stronger cross-linking as determined in this study compared to our previous study probably resulted in higher negative charge density in the microparticles, which might have improved siRNA binding, release, and presentation to the adhering cells. The influence of the DEED modification of the cross-linker, however, needs further systematic investigation.

The combination of chordin siRNA and BMP-2 stimulation led to significantly elevated ALP expression and enzyme activity, which is a relevant marker for in vivo bone formation in stem cells [47]. The cross-linked microparticles mineralized in an osteogenic medium after 8–9 days, both in the presence and in the absence of cells. The ability for mineralization and the strong silencing effect clearly distinguishes the here presented cGM from previously published data [34]. Compared to the former study, cGM showed a significantly higher degree of cross-linking and higher incorporation of the cross-linker oPNMA^+DEED^ in the presence of TEA as base. The existing carboxyl groups in gelatin and the incorporated carboxyl groups of the cross-linker enhanced the negative charges in the microparticles. These presumably support calcium phosphate nucleation, either directly or via the adsorption of mineralizing osteogenic proteins, such as osteopontin or bone sialoprotein [35]. In contrast to our previous study [34] where we used cGM that did not mineralize in the absence of cells, we could not measure any differences in the calcium content in the different groups after 14 or 21 days. Calcium content, however, was strongly increased in all groups compared to the previous study with cGM of a lower cross-linking degree [34]. This result supports our hypothesis that the electrostatic conditions associated with the amount of incorporated cross-linker are strongly related to the intensity and rate of mineralization.

### 4.2. Chordin Silencing Influences Mineral Composition in Microtissues

The ability of the cGM for biomineralization could positively influence osteogenic differentiation [48,49,50]. In cell-independent mineralization, as well as the availability of calcium and phosphate, the chemical composition of the environment such as pH and CO_2_, plays a decisive role regarding the morphology, water content, element composition, structure, and particle size of precipitated minerals [51,52,53]. During the biologically controlled mineralization, cells also influence nucleation, growth, morphology, and the final location of the deposited mineral [51]. 

In chordin-silenced and BMP-2-stimulated microtissues, mineralized cGM appeared morphologically different from non-coding siRNA and non-treated cGM controls. The surface of the particles was smoother, possibly indicating nano-structured mineral in contrast to micron-structured mineral formation in controls. This may be explained by chordin silencing that led to increased bioavailability of endogenously produced and exogenously supplemented BMP-2 in hMSC, which led to a downstream increase in ALP expression and enhanced osteogenic differentiation [10,32,33]. Li et al. also reported differences in mineral structure depending on ALP activity for chitosan hydrogels [54]. They described that precipitated pure glycerophosphate shows rough micron-scaled structures, whereas mineral formation by ALP-cleaved glycerophosphate showed nano-scaled structures. Similarly, Öfkeli et al. reported gelatin hydrogel biomineralization with simulated body fluid and also observed micron-sized HA-particles in the gels [55]. We therefore speculate that ALP may play a special role in our system. The enzyme catalyzes the hydrolysis of organic phosphate to release free inorganic phosphate providing the chemical precursors for mineral deposition [53,56,57]. In contrast to former studies [10,32], we did not find a reduced proliferation rate in chordin-silenced samples compared to the non-treated control.

We assume that due to the mineralization of the particles, the biomechanical properties of the cGM also changed during culture. It is known from the literature that the shape, stiffness, and geometry of microparticles could influence the fate and functional behavior of cells [58]. However, an analysis was beyond the scope of this paper. A synergistic effect of biochemical signaling of the cells, the associated increased ALP activity, and the influence of the material itself is possible.

### 4.3. Microtissues in a Supplement-Free Co-Culture of hMSC and PBMC

In order to mimic the influence of pre-differentiated microtissues on bone tissue formation, we used an established in vitro co-culture system of osteoblasts and osteoclasts [39,40]. Chordin-silenced and BMP-2-treated microtissues stimulated the differentiation of stem cells in the co-culture, which would be indicated by enhanced ALP activity. Recent studies have shown that the gene expression of the osteoblast transcription factors Runx2 and osterix are dependent on ALP activity and thereby responsible for osteoprogenitor differentiation [59]. In addition, ALP catalyzes the dephosphorylation of phosphorylated osteopontin (p-OPN) [60]. Furthermore, the chordin-silenced and BMP-2-stimulated microtissues led to upregulated OPG concentrations in the co-culture. OPG is secreted by osteoblasts whether or not osteoclasts are present [61] and represents, together with receptor activator of nuclear factor (NF-kappaB) ligand (RANKL), an important paracrine regulator of osteoclastogenesis. Osteoblast-produced factors could directly activate (macrophage colony-stimulating factor/M-CSF, RANKL) [62,63] or inhibit (OPG) [41,42] the osteoclastogenesis.

The presence of high OPG level in the co-culture inhibits RANKL-binding to its receptor and therefore the maturation of PBMC to osteoclasts. The measurable TRAP activity and microscopic images in a control plate (data not shown) indicate the presence of a low number of osteoclasts in the co-culture.

Nevertheless, there are some unanswered questions that we have not yet been able to clarify.

Could the differently pre-treated microtissues alone stimulate the undifferentiated stem cells to the osteogenic lineage, which could be shown by an increased ALP and OPG? Birmingham et al. reported the differentiation of stem cells only by the regulation of osteoblasts or osteocytes [64]. The paper described that higher differentiated osteocytes accelerated the differentiation compared to less-differentiated osteoblasts, which supports our hypothesis.

Could the particles in the differentiated microtissues adsorb BMP-2 and thus influence the unstimulated hMSC? It has been shown that BMP-2 could induce increased levels of OPG in osteoblast-like cells [61,65]. 

Are the measured ALP and OPG levels caused by the microtissues or by the stimulated hMSC in the co-culture? An interesting study from Granero-Moltό et al. showed that transplanted MSCs contribute to fracture healing by expressing BMP-2 [66], which could also be an explanation for the increasing ALP and OPG level in our results. Due to the complex interrelationships, we cannot explain which paracrine effects trigger the stimulation of the stem cells, yet. However, we suspect that our results reflect synergistic effects.

Several studies described problems due to interactions of positively charged nucleic acid delivery systems for gene therapy with blood components [67], negative effects to the immunoglobulin production [68], or cytotoxicity [69,70]. We suppose that the release of the siRNA to the microtissue forming cells in vitro provides very defined local effects that at the time of implantation, are not expected to induce any side effects via siRNA. The transfection with chordin siRNA and stimulation with BMP-2 lead to a faster differentiation of hMSC in the microtissues. These microtissues influence cells in their direct vicinity. Nevertheless, for clarity concerning therapeutic effects as well as possible side effects, in vivo experiments are needed.

The microtissues are designed to be introduced into a bone defect in vivo either directly or via a matrix. In our in vitro studies, the microtissues remained stable up to the maximum duration of the experiment of 28 days. In any case, this time seems to be sufficient to stimulate the surrounding cells and to serve as a starting point for mineralization, as we have shown here and in the previous work [32]. Bone is a highly dynamic tissue that is constantly being remodeled by osteoblasts and osteoclasts. We suspect that the mineralized microtissues will be replaced by the body’s own tissue after several weeks to months [71]. 

## 5. Conclusions

We presented mineralizing cGM, which form microtissues with hMSCs. Via siRNA loading of cGM, we showed highly efficient silencing of chordin and, in consequence, strongly increased ALP activity, supported by supplementation with BMP-2. Chordin-silenced microtissues precultured with BMP-2 were able to mediate osteoanabolic effects in co-cultures of hMSC and hPBMC. This may be an indication that microtissues from autologous hMSC may be able to mimic bone autografts in bone defects.

## Figures and Tables

**Table 1:** TaqMan™ Gene Expression assays.

Symbol	Gene Name	Assay ID
ALPL	Alkaline phosphatase activity, liver/bone/kidney	HS01029144_m1
CHRD	Chordin	HS00415315_m1
RPLP0	60S acidic ribosomal protein P0	HS99999902_m1

**Table 2:** Average particle size of dry and swollen particles.

Particle State	D [4,3]
dry particles	79.15 ± 10.68 µm
swollen particles	137.67 ± 18.77 µm

**Table 3:** Proliferation rates (increase DNA in ng per day) in microtissues from siRNA-loaded cGM and hMSC in an osteogenic medium with or without BMP-2 (ctr: non-treated control, nc: non-coding siRNA, chrd: chordin siRNA).

Sample	Proliferation Rate ng/Day	R^2^
ctr	2.66	0.9938
nc	4.52	0.9837
chrd	3.21	0.9953
ctr^+BMP-2^	0.61	0.2336
nc^+BMP-2^	2.10	0.7228
chrd^+BMP-2^	0.70	0.972

## Data Availability

On request, the raw data can be sent by email.

## Supplementary Materials

The following supporting information can be downloaded at: https://www.mdpi.com/article/10.3390/pharmaceutics14030548/s1, Figure S1: Representative images (phase contrast microscopy) of microtissue mineralization with and without siRNA or/and BMP-2; Figure S2: Phase contrast images of cGM control wells after 7, 14, and 21 days in an osteogenic medium in absence of cells.
