# Supplementary material for: Mineralizing Gelatin Microparticles as Cell Carrier and Drug Delivery System for siRNA for Bone Tissue Engineering
Source: Pharmaceutics. 2022 Feb 28;14(3):548. doi: 10.3390/pharmaceutics14030548 (PMC8949427; doi:10.3390/pharmaceutics14030548)
Supplement: Supplementary file 1 [file pharmaceutics-14-00548-s001.zip › pharmaceutics-1586391-supplementary.pdf]

pharmaceutics

Supplementary Materials: Mineralizing Gelatin Microparticles as Cell Carrier and Drug Delivery System for siRNA for Bone Tissue Engineering

Sandra Hinkelmann, Alexandra H. Springwald, Sabine Schulze, Ute Hempel, Franziska Mitrach, Christian Wölk, Michael C. Hacker and Michaela Schulz-Siegmund

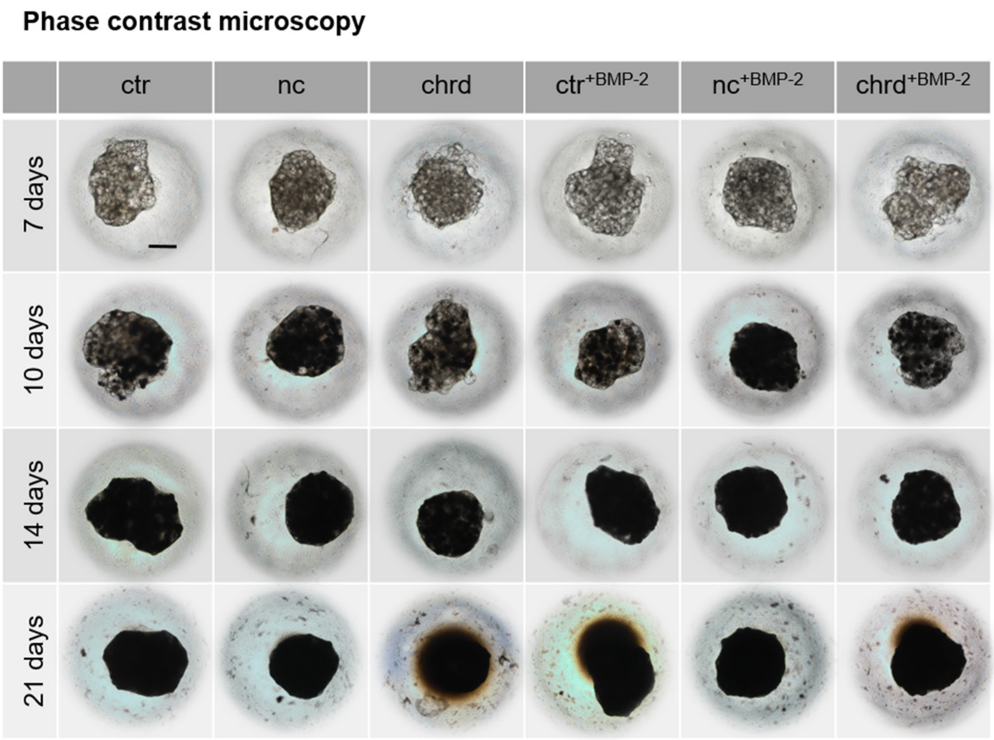

Figure S1: Representative images (phase contrast microscopy) of microtissue mineralization with and without siRNA or/and BMP-2.

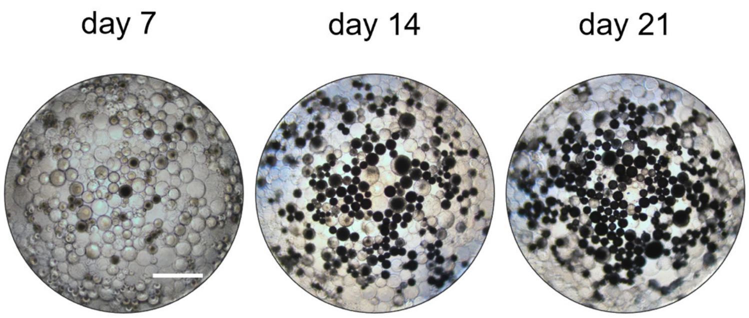

Figure S2: Phase contrast images of cGM control wells after 7, 14, and 21 days in an osteogenic medium in absence of cells.
